# Omission of Subtitle

**DOI:** 10.1001/jamahealthforum.2022.3905

**Published:** 2022-10-14

In the Viewpoint titled “Strengthening Primary Care to Improve Health Outcomes in the US—Creating Oversight to Address Invisibility,”^1^ published online on September 2, 2022, the subtitle had been omitted. This article was corrected online.
